# Identification of Common Prognostic Gene Expression Signatures with Biological Meanings from Microarray Gene Expression Datasets

**DOI:** 10.1371/journal.pone.0045894

**Published:** 2012-09-21

**Authors:** Jun Yao, Qi Zhao, Ying Yuan, Li Zhang, Xiaoming Liu, W. K. Alfred Yung, John N. Weinstein

**Affiliations:** 1 Department of Neuro-Oncology, The University of Texas M. D. Anderson Cancer Center, Houston, Texas, United States of America; 2 Department of Biostatistics, The University of Texas M. D. Anderson Cancer Center, Houston, Texas, United States of America; 3 Department of Bioinformatics and Computational Biology, The University of Texas M. D. Anderson Cancer Center, Houston, Texas, United States of America; 4 School of Public Health, The University of Texas – Houston Health Science Center, Houston, Texas, United States of America; 5 Department of Neurosurgery, John Hopkins University, Baltimore, Maryland, United States of America; Mount Sinai School of Medicine, United States of America

## Abstract

Numerous prognostic gene expression signatures for breast cancer were generated previously with few overlap and limited insight into the biology of the disease. Here we introduce a novel algorithm named SCoR (***S***urvival analysis using ***Co***x proportional hazard regression and ***R***andom resampling) to apply random resampling and clustering methods in identifying gene features correlated with time to event data. This is shown to reduce overfitting noises involved in microarray data analysis and discover functional gene sets linked to patient survival. SCoR independently identified a common poor prognostic signature composed of cell proliferation genes from six out of eight breast cancer datasets. Furthermore, a sequential SCoR analysis on highly proliferative breast cancers repeatedly identified T/B cell markers as favorable prognosis factors. In glioblastoma, SCoR identified a common good prognostic signature of chromosome 10 genes from two gene expression datasets (TCGA and REMBRANDT), recapitulating the fact that loss of one copy of chromosome 10 (which harbors the tumor suppressor PTEN) is linked to poor survival in glioblastoma patients. SCoR also identified prognostic genes on sex chromosomes in lung adenocarcinomas, suggesting patient gender might be used to predict outcome in this disease. These results demonstrate the power of SCoR to identify common and biologically meaningful prognostic gene expression signatures.

## Introduction

Multiple prognostic gene expression signatures have been identified in the past decade for breast cancers [1]–[7] and some are now used to predict patient outcome and assist treatment decisions. For example, the NKI 70-gene signature [1], [2] (Mammaprint, Agendia) and OncotypeDX signature [8] (Recurrence Score, Genomic Health) became commercially available and commonly used in the clinics. Yet, two questions remain. First, there is little overlap among numerous prognostic signatures generated from different studies [9]. Second, most signatures generated by approaches without using prior knowledge from gene ontology failed to provide us with clear biological meanings as why these prognostic signatures (e.g. the 70-gene signature) may affect patient outcome. As a result, the clinical application of such prognostic signatures is still under debate [10], [11].

A study by Liat Ein-Dor et al pointed out that the NKI 70-gene signature was not a unique gene expression signature and many other 70-gene signatures could be generated from the same dataset and behave similarly in predicting breast cancer outcome [9], suggesting previous methods were not reaching the real prognostic signature but merely finding surrogates. Similarly, Michels et al found the prognostic gene lists generated from microarray studies were highly unstable and strongly dependent on the selection of patients in the training sets [12]. These findings may explain why different prognostic signatures did not match one another and why they did not provide biological insight into the disease. Although lack of overlap in signature genes does not necessarily mean there is no commonality in captured biology [13], [11], identification of authentic gene expression signatures that are most related to the causal genes of the diseases is always desired.

In this study, we developed a novel algorithm named SCoR (***S***urvival analysis using ***Co***x proportional hazard regression and ***R***andom resampling) and demonstrated that using resampling and clustering techniques could help reduce overfitting artifacts and produce prognostic gene expression signatures with apparent biological functions matching the pathologic phenotypes of the diseases. By applying SCoR to a panel of breast cancer gene expression datasets, we repeatedly found overexpression of the cell division cycle genes a common poor prognostic signature in breast cancers. Likewise, we identified increased expression of chromosome 10 genes (indicative of absence of PTEN LOH on chromosome 10) a good prognostic signature for glioblastoma patients in two different datasets. In addition, we demonstrate that SCoR has the ability to pick only a few genes on chromosome Y and X in a lung adenocarcinoma dataset, suggesting that patient gender may be used as a prognostic parameter in lung adenocarcinoma patients.

## Methods

### Public Gene Expression Datasets and Data Processing

Breast cancer datasets were downloaded from the NCBI GEO website (http://www.ncbi.nlm.nih.gov/geo) and for the NKI-295 dataset from http://microarray-pubs.stanford.edu//wound_NKI/explore.html. Glioblastoma datasets were downloaded from TCGA and RAMBRANDT websites (http://tcga-data.nci.nih.gov/tcga/findArchives.htm and https://caintegrator.nci.nih.gov/rembrandt). Lung adenomcarcinoma datasets were downloaded from the NCBI GEO website and for the DCC2008-MI dataset downloaded from website listed by the authors [14]. A brief summary of the datasets used in this study is listed in supplementary Table S1. All Affymetrix based CEL files from one dataset were RMA normalized using the “affy” package from the R software (available at http://www.bioconductor.org and http://www.r-project.org). Normalized gene copy numbers for TCGA glioblastoma samples were generated from MSKCC array CGH level 3 data. Briefly, log2 values representing 1N, 2N, and 3N chromosomal segments for each tumor were determined and used to scale level 3 log2 values to the same level with log2 values of −1, 0, and 0.585 representing 1N, 2N, and 3N chromosomal segments, respectively. For PTEN copy number calls, PTEN gene with log2 values below −0.7 were called as copy loss, and below −1.5 called as deletion, otherwise called as normal.

### SCoR Analysis

Normalized micoarray data were filtered to remove low variance probesets based on median absolute deviation (MAD) with the bottom 20% of probesets discarded (∼30% for HT-HG_U133A platform, which comprised of a background peak). Survival analysis was performed using Cox proportional hazards regression model (coxph function from the R “survival” package) on each probeset on a randomly chosen subset of samples representing 75% of all patients. Any probeset with a coxph *p* value of <0.01 was considered positive. The above procedure was repeated 400 rounds without replacement of sample subsets and results merged together to calculate frequency for each probeset to be positive among all runs. Positive probesets with frequency above a threshold (typically 75%, subjected to change) were collected as top candidates. All parameters (e.g., percentage of patient subset, coxph *p* value cutoff, and frequency cutoff) could be tuned for a particular dataset based on the size and quality of the dataset. An unsupervised clustering was done using Cluster and TreeView software from Michael Eisen’s lab (http://www.eisenlab.org/eisen) on gene expression data from top candidate probesets from all patient samples. The parameters used were hierarchical cluster, Spearman rank correlation, and average linkage clustering.

### Patient Stratification and Kaplan Meier Analysis

Gene expression data for a particular prognostic signature is collected and normalized by first median centering and then dividing by MAD for each probeset. A multi-gene score was calculated for each patient as the averaged sum of normalized expression values from good prognosis probesets minus those from the poor ones, similar to the Relapse Score and Gene expression Grade Index (GGI) described before [5], [6]. The formula is: Multi-Gene Score  =  ∑x_i_ − ∑x_j_, where i and j include good and poor prognostic gene probesets, respectively. The patients were divided into two groups mathematically (with high and low scores) based on their multi-gene score values using the “kmeans” function from the R software. Kaplan Meier plots were drawn using the “survival” package from the R software.

### Gene Enrichment in Prognostic Gene Expression Signature

To examine whether a particular gene set was enriched in SCoR identified prognostic gene list, we performed a Fisher exact test on number of genes of particular function (e.g. chromosome 10 genes) within the prognostic gene list compared to those found in background (all other genes not in the prognostic gene list). The enrichment fold was calculated as percentage of genes of particular function inside the prognostic list over that in the background.

## Results

### SCoR Analysis Outline

**Figure 1:**
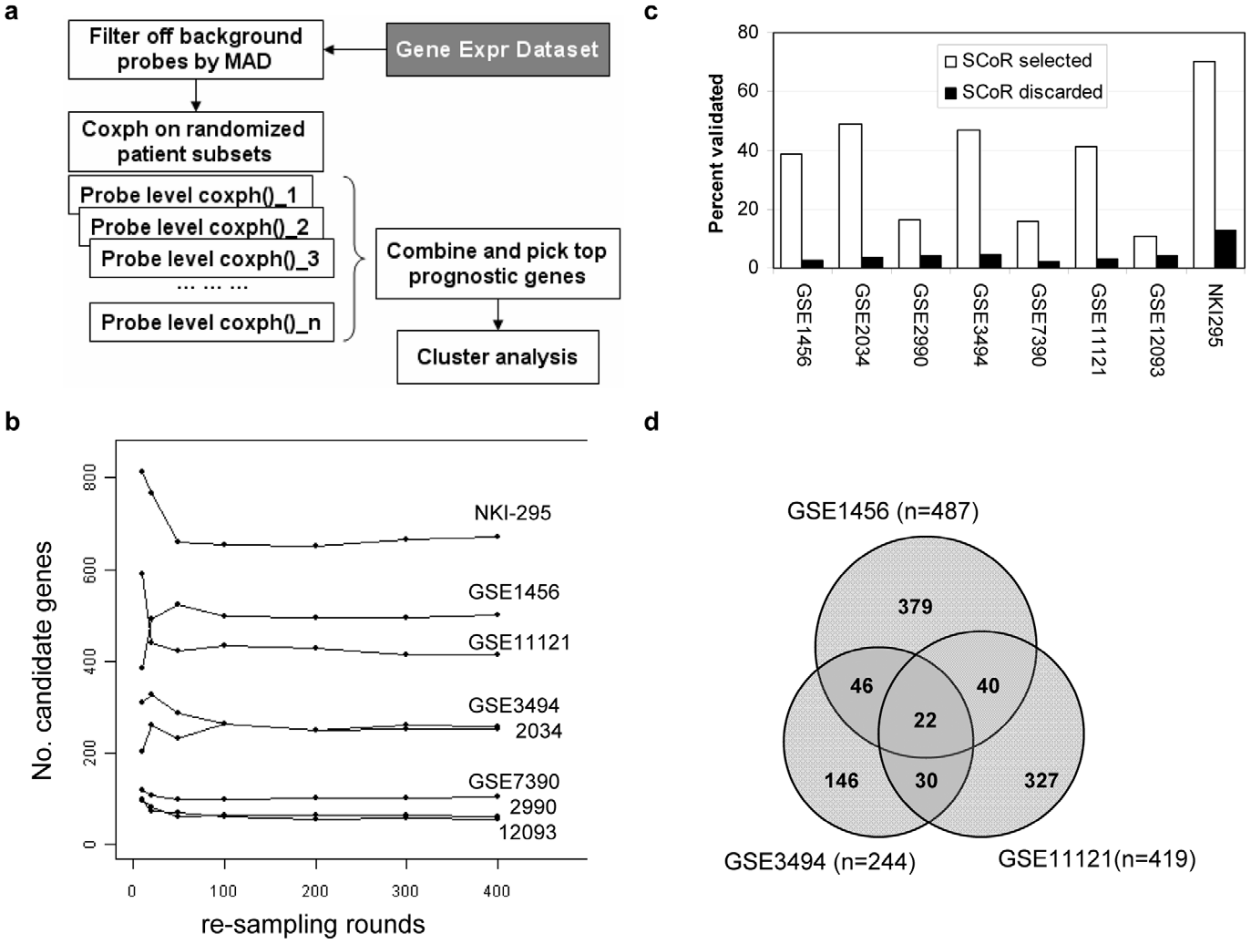
Outline of SCoR procedure and output results on breast cancer datasets. (a) outline of SCoR procedure (b) number of candidate prognostic genes generated by SCoR using different resampling rounds (c) percent of candidate prognostic probesets passed the internal validation using remaining samples not used in SCoR (d) Venn diagram showing overlapping genes found by SCoR in three breast cancer datasets.

We developed a method named **SCoR** (**S**urvival analysis using **Co**x proportional hazard regression and **R**andom resampling) on top of the Cox proportional hazard regression method (Coxph) that is commonly used in survival analysis (Fig. 1a). Briefly, after filtering off background probesets using an arbitrarily set median absolute deviation cutoff (see materials and methods), we performed a univariant Coxph test for each probeset on a subset of patient data, which is comprised of typically 75% of all patients. Probesets passed a Coxph *p* value cutoff (default<0.01) were collected as candidate prognostic probesets. This procedure was repeated up to 400 times with the patient subset randomly reset for each new run with no replacement. Results from all individual runs were then merged and probesets passed an arbitrarily set frequency threshold were selected as top prognostic candidate probes (typically at 75%, i.e., any probesets passed 3 out 4 times in all random Coxph tests were considered as prognostic). As shown in Fig. 1b, the number of top prognostic candidate genes selected became saturated when resampling rounds reached about 200. Therefore, most of our analyses were based on 200 resampling runs. We also performed an internal validation procedure on Coxph identified candidate probesets in the remaining 25% or so patients that were not directly used in SCoR runs. The overall percentage of probesets passed this validation procedure was low and usually less than 10%. However, this percentage was increased to >40% in probesets that were selected by SCoR, arguing that the SCoR procedure had the ability to effectively enrich real target prognostic genes (Fig. 1c). Top candidate prognostic genes were further analyzed by unsupervised clustering of their expression from all patient samples to identify any groups of genes having similar expression patterns, hence might function in the same pathway to affect tumor survival.

### SCoR Identified a Common Poor Prognostic Gene Expression Signature Comprised of Cell Division Genes from Different Breast Cancer Datasets

We applied SCoR to eight breast cancer gene expression datasets (supplementary table S1). This yielded varied numbers of candidate prognostic genes, from as low as 53 genes at a SCoR frequency cutoff of 50% in GSE2990, to as high as 651 genes at the 90% frequency cutoff in NKI-295 (Table 1, Fig. 1b). Remarkably, significant overlap between results from different datasets could be observed. For example, up to 40% of candidate genes found in GSE3494 could also be found in SCoR results from either GSE1456 or GSE11121 (Fig. 1d). A top overlap signature was therefore compiled from all SCoR results (Table 1), comprised of 85 genes, each having been found at least 3 times in any of the eight datasets. Notably, the majority of these genes are associated with poor prognosis, and 58 out of 85 genes had known function in direct regulation of cell division.

**Table 1:** top overlapping prognostic genes identified from different breast cancer datasets.

	GSE 1456	GSE 2034	GSE 2990	GSE 3494	GSE 7390	GSE 11121	GSE 12093	NKI 295				
*SCoR freq cutoff*	75	75	50	75	50	75	50	90				
*no. candidate prognostic genes*	495	249	53	247	101	429	63	651				
*genes in top 85 overlapping genes*	65	29	5	54	7	58	12	61				
**no.**	**top overlap genes**	**GSE 1456**	**GSE 2034**	**GSE 2990**	**GSE 3494**	**GSE 7390**	**GSE 11121**	**GSE 12093**	**NKI 295**	**freq.**	**prog.**	**function**
*1*	CREBL2	1			1		1		1	4	good	tumor suppressor
*2*	CRIM1	1			1				1	3	good	
*3*	SPARCL1	1			1				1	3	good	
*4*	GLTSCR2	1				1			1	3	good	tumor suppressor
*5*	PDS5B	1				1			1	3	good	tumor suppressor
*6*	CD302	1			1		1			3	good	
*7*	FBLN1	1			1		1			3	good	tumor suppressor
*8*	KIAA0494	1			1		1			3	good	
*9*	N4BP2L1	1			1		1			3	good	
*10*	SPTAN1		1		1		1			3	good	
*11*	CCNB2	1	1		1		1		1	5	poor	proliferation
*12*	NUSAP1	1	1		1		1		1	5	poor	proliferation
*13*	PRC1	1	1		1		1		1	5	poor	proliferation
*14*	RACGAP1	1	1		1		1		1	5	poor	proliferation
*15*	STMN1	1			1		1	1	1	5	poor	proliferation
*16*	CDKN3	1	1		1			1	1	5	poor	proliferation
*17*	KIF23	1			1		1		1	4	poor	proliferation
*18*	MELK	1			1		1		1	4	poor	proliferation
*19*	MYBL2	1			1		1		1	4	poor	proliferation
*20*	PTTG1	1			1		1		1	4	poor	proliferation
*21*	RAD51	1			1		1		1	4	poor	proliferation
*22*	SPAG5	1			1		1		1	4	poor	proliferation
*23*	UBE2C	1			1		1		1	4	poor	proliferation
*24*	BUB1	1	1				1		1	4	poor	proliferation
*25*	CCNA2	1	1				1		1	4	poor	proliferation
*26*	CENPF	1	1				1		1	4	poor	proliferation
*27*	DLGAP5	1	1				1		1	4	poor	proliferation
*28*	H2AFZ	1	1				1		1	4	poor	
*29*	NEK2	1	1				1		1	4	poor	proliferation
*30*	SQLE	1					1	1	1	4	poor	
*31*	GINS2	1	1		1				1	4	poor	proliferation
*32*	RRM2	1	1		1				1	4	poor	proliferation
*33*	CIAPIN1	1			1	1			1	4	poor	
*34*	NCAPG	1			1		1	1		4	poor	proliferation
*35*	MLF1IP	1	1		1	1				4	poor	proliferation
*36*	CTTN	1		1		1		1		4	poor	proliferation
*37*	ESPL1		1		1		1		1	4	poor	proliferation
*38*	ZWINT				1		1	1	1	4	poor	proliferation
*39*	CCNE2		1				1	1	1	4	poor	proliferation
*40*	CDC25A	1					1		1	3	poor	proliferation
*41*	EXO1	1					1		1	3	poor	proliferation
*42*	FEN1	1					1		1	3	poor	proliferation
*43*	FOXM1	1					1		1	3	poor	proliferation
*44*	KIF2C	1					1		1	3	poor	proliferation
*45*	MCM6	1					1		1	3	poor	proliferation
*46*	NCAPH	1					1		1	3	poor	proliferation
*47*	ACOT7	1			1				1	3	poor	
*48*	C16orf61	1			1				1	3	poor	
*49*	C20orf24	1			1				1	3	poor	
*50*	CCNB1	1			1				1	3	poor	proliferation
*51*	GOT1	1			1				1	3	poor	
*52*	TACC3	1			1				1	3	poor	proliferation
*53*	DTL	1	1						1	3	poor	proliferation
*54*	KPNA2	1	1						1	3	poor	proliferation
*55*	SHCBP1	1	1						1	3	poor	proliferation
*56*	C16orf80	1				1			1	3	poor	
*57*	COG4	1		1					1	3	poor	proliferation
*58*	SNRPA1	1		1					1	3	poor	
*59*	CDC20	1			1		1			3	poor	proliferation
*60*	DDX39	1			1		1			3	poor	
*61*	DHFR	1			1		1			3	poor	
*62*	KIF20A	1			1		1			3	poor	proliferation
*63*	CIAO1	1	1				1			3	poor	proliferation
*64*	KIAA0101	1	1				1			3	poor	proliferation
*65*	TOP2A	1					1	1		3	poor	proliferation
*66*	TIMM17A	1	1		1					3	poor	
*67*	PPFIA1	1			1	1				3	poor	
*68*	APRT	1		1	1					3	poor	
*69*	NOL3	1	1	1						3	poor	proliferation
*70*	EBP				1		1		1	3	poor	proliferation
*71*	H2AFX				1		1		1	3	poor	proliferation
*72*	KIFC1				1		1		1	3	poor	proliferation
*73*	MAD2L1				1		1		1	3	poor	proliferation
*74*	NDUFS6				1		1		1	3	poor	
*75*	SNRPC				1		1		1	3	poor	
*76*	SPC25				1		1		1	3	poor	proliferation
*77*	AURKA		1				1		1	3	poor	proliferation
*78*	TMPO		1				1		1	3	poor	proliferation
*79*	GTSE1						1	1	1	3	poor	proliferation
*80*	CENPM				1			1	1	3	poor	proliferation
*81*	GINS1		1		1		1			3	poor	proliferation
*82*	KIF11		1		1		1			3	poor	proliferation
*83*	TAF11		1		1		1			3	poor	
*84*	CDC2				1		1	1		3	poor	proliferation
*85*	MKI67				1		1	1		3	poor	proliferation

“1” denotes that the gene is found by SCoR in this dataset. (a), Kaplan Meier plot of patient survival stratified by gender in stage II/III lung adenocarcinomas (top) or stage I lung adenocarcinomas (bottom) in GSE4716. (b), same as (a) except using data from GSE13213. (c), Kaplan Meier plot of lung adenocarcinoma recurrence stratified by patient gender using data from GSE25326. (d), Fisher exact test of patient survival vs. gender using a 2×2 contingency table and data from GSE28582.

**Figure 2:**
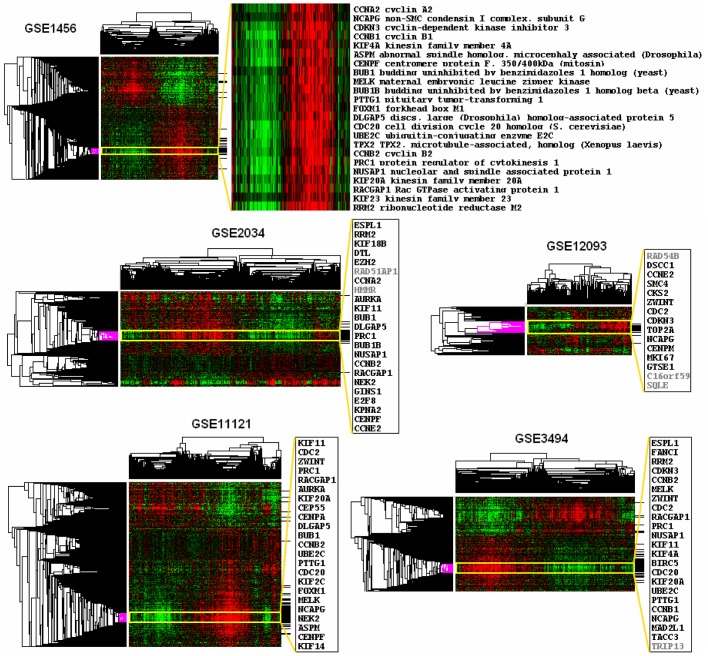
Identification of a common poor prognostic gene expression signature in breast cancer. Top, cluster heatmap of gene expression using SCoR generated prognostic genes from GSE1456 dataset with a blowup of genes inside the center of the poor prognosis gene cluster (yellow box), genes matching top overlap genes from Table 1 were marked in black lines on the right side of the heatmap. Bottom panels, similar to top but only show the gene symbols from the center cluster. All genes involved in cell division cycle regulation are in black, otherwise are in grey.

**Figure 3:**
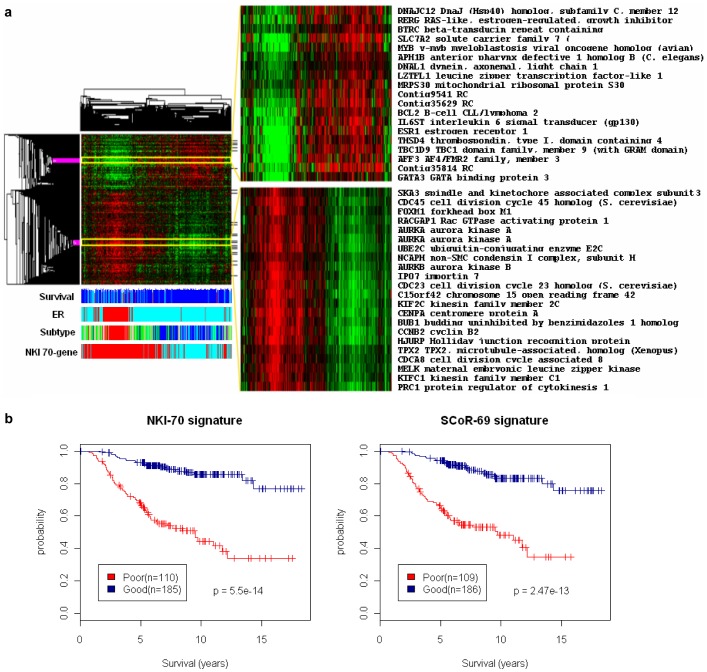
Comparision of NKI 70-gene signature with SCoR derived 69-gene signature. (a), cluster heatmap of gene expression using SCoR generated prognostic probes from NKI-295 dataset with blowup views of genes inside the center of poor and good prognosis gene clusters, probes matching the NKI 70-genes were marked in black lines on the right side of the heatmap. Patient survival is marked in blue (>5yr), red (dead in 2yr), or otherwise cyan. ER status is marked in red (ER-) or cyan (ER+). Molecular subtypes are marked in blue (luminal A), green (luminal B), red (basal), ERBB2 (pink), or cyan (normal-like). NKI 70-gene prediction is marked in red (poor prognosis) or cyan (good prognosis). (b), Kaplan Meier plots of patient survivals stratified by either the NKI 70-gene signature or the SCoR 69-gene signature.

When unsupervised clustering of candidate SCoR genes was performed for each dataset, we observed a clear enrichment of top overlap cell division cycle (CDC) genes at the center of poor prognostic gene clusters (Fig. 2). Expression levels of these genes are highly correlated to one another, suggesting that these genes might be under a same transcriptional control (e.g. FOXM1, which is also among the top overlap gene list). Poor prognostic signatures generated from the cluster centers of different datasets again displayed a uniform composition of CDC genes. In GSE1456 and GSE11121, based on gene ontology analysis and literature search, 100% genes within the selected core signatures are involved in cell cycle regulation (Fig. 2). These signatures were highly similar, though not exactly the same. This similarity could be found in six out of eight datasets (Fig. 2 and 3, Table 1), and can be appreciated even when a low number of SCoR candidates were identified (e.g. Fig. 2, GSE12093). Thus, we demonstrate that a common prognostic gene expression signature could be identified from different datasets using one identical method (SCoR) in a completely unbiased manner.

### Comparing SCoR Generated Prognostic Signature with NKI 70-gene Signature and Cross Validation of CDC Signatures

To compare our SCoR candidates with the renowned NKI 70-gene signature [1], [2] (MammaPrint®), we paid more attention to results generated from the NKI-295 dataset, which included samples used to generate the NKI 70-gene signature. Notably, data from this dataset were generated using a rather old microarray platform (Rosetta human 25K). Also, the NKI 70-gene signature was generated using 78 samples having extreme survival length and metastasis status in a supervised manner, while we used all 294 patient samples and performed SCoR analysis in a unsupervised manner. Still, we were able to identify the CDC gene signature at the center of the poor prognostic gene cluster (Fig. 3a). In addition, we identified ER, Bcl-2, Myb, and GATA3 at the center of the good prognostic gene cluster, which matched perfectly with the known clinical and pathologic roles of these molecules in breast cancer [15]–[18]. This particular distribution pattern of established prognostic factors such as proliferation genes, ER, and Bcl-2 in the SCoR cluster heatmap argues that survival analysis may yield both “real” prognostic genes as well as “passengers” whose expression closely matched the expression of real ones. This is a plausible explanation why real signatures may reside in the center of the cluster as surrounding “passengers” are mathematical by-products of the analysis. Based on this idea, we constructed a 69-gene signature using core cluster genes from both poor and good prognostic gene clusters based on SCoR results from NKI-295 (37 CDC genes plus 32 genes inside the ER/Myb/Bcl-2 cluster). Among the top 651 probes identified by SCoR, 25 matched those from the NKI 70-gene signature (marked in Fig. 3). Only 3 NKI-70 genes were found inside the core CDC gene signature (CENPA, MELK, PRC1) and none inside the ER/Myb/Bcl-2 core cluster (Fig. 3a). Using a multigene score algorithm (see methods and materials), we stratified patients based on either NKI 70-gene signature or our SCoR derived 69-gene signature to compare their abilities to predict clinical outcome. The result was quite comparable from two signatures, with an overall agreement of 84% on patient stratification (Fig. 3b). However, the 69-gene signature bears more biological meanings and clinical relevance. The SCoR cluster effectively separated breast cancer patients into groups enriched in ER-negative, basal and luminal B tumors versus ER-positive and luminal A tumors (Fig. 3a). When applied to other breast cancer datasets, the 69-gene signature overall performed slightly better than the 70-gene signature (supplementary Table S2). We further cross validated the core CDC signatures generated from SCoR analysis on individual datasets (supplementary Table S2). This demonstrated that CDC signatures (26 or 27-gene) from GSE1456, 2034, and 3494, though not exactly the same, can effectively separate patients into good and poor prognostic groups in all breast cancer datasets tested, even in GSE2990 and 7390 where SCoR failed to identify such CDC signatures as prognostic gene expression signatures. The top overlap 85-gene signature was also validated in all datasets and seemed to have the best performance among all signatures.

### SCoR Identified T/B Cell Markers as Favorable Prognostic Factors in Highly Proliferative Breast Cancers

We further used CDC gene signature to stratify patients into high and low proliferation groups and performed SCoR analysis on different patient populations. This identified in GSE2034 dataset both B- and T-cell specific markers as favorable prognostic genes in highly proliferative breast cancers. SCoR could also independently identify a similar B-cell signature in GSE11121 (supplementary Fig. S1). Both GSE2034 and GSE11121 contain lymph node negative patients. Signatures generated from GSE2034, especially the T-cell signature, could be cross validated in other datasets such as GSE3494, GSE7390, and GSE11121, and had a tendency to discriminate patients survival in GSE1456 and GSE12093 (supplementary Table S3). These signatures were not validated in NKI295 dataset when patients were stratified by a CDC signature. However, when we performed SCoR on ER-negative patients from NKI295, which composed mainly of highly proliferative basal and HER2 subtype breast cancers, we also identified a T-cell signature in patients with better survival (supplementary Fig. S1). The overlap among T- and B-cell signatures generated from GSE2034, GSE11121, and NKI295 were not significant on the gene level except for the presence of immunoglobulin genes (IGH/IGL genes). However, on the functional level, many of these SCoR generated genes clearly pointed to B-cell and T-cell involvement. The ability of SCoR to identify common theme prognostic signatures in this sequential analysis adds proof to the validity of our approach. These findings are supported by several reports from the literature [19], [7], [20].

### SCoR Identified a Good Prognostic Gene Expression Signature Comprised of Chromosome 10 Genes in Glioblastomas

**Figure 4:**
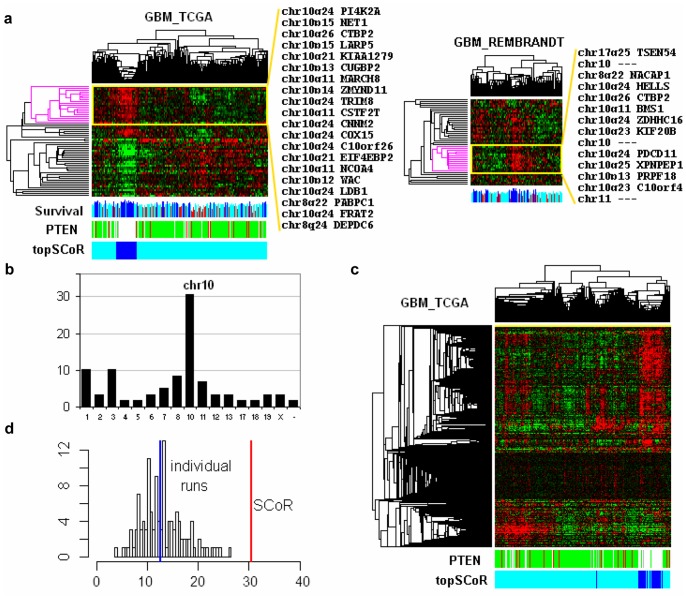
Identification of a common good prognostic gene expression signature in glioblastomas. (a) cluster heatmaps of gene expression using SCoR generated prognostic genes from TCGA and REMBRANDT datasets with annotated genes and chromosomal locations on selected good prognostic gene clusters. Patient survivals were plotted in blue (>600 days) or red (<180 days) representing top and bottom quartile survivals. PTEN copy numbers are plotted in white, green, and red, representing normal copy number, copy loss, and deletion, respectively. “topSCoR” tumors, representing a group of samples having highest expression from good prognostic genes, were marked in dark blue. (b) distribution of SCoR generated prognostic genes on all chromosomes, plotted in percentage. (c) cluster heatmap using 791 chromosome 10 probesets and 188 glioblastoma patients. PTEN copy numbers were plotted in white, green, and red, representing copy number calls of 2N, 1N, and 0N, respectively. “topSCoR” tumors marked positions of same tumors found by SCoR in panel (a). (d). histogram of percentage of chromosome 10 genes found in individual SCoR runs, blue line marked the median of these percentages and red line marked the percentage of chromosome 10 genes found by SCoR.

We next extended our study to glioblastoma, a type of malignant brain tumor with rather poor prognosis among all cancers. In two glioblastoma datasets (TCGA and REMBRANDT), SCoR repeatedly found enrichment of chromosome 10 genes within prognostic candidates, which constituted nearly one third of all prognostic candidates identified (8.4-fold enrichment and Fisher exact test *p* value 1.45e-12 for TCGA samples, Fig. 4A, B). However, there was little overlap between these two sets of chromosome 10 genes (only one gene overlap, CTBP2). Also, the locations of these genes are not restricted to any particular chromosomal locus but rather scattered on the whole chromosome, suggesting an overall expression level change on chromosome 10. Since it is known that the tumor suppressor gene PTEN (located on chr10q23) is frequently inactivated in glioblastomas through mechanisms including partial or entire loss of chromosome 10 [21] we reasoned that increased expression of genes on chromosome 10 might be an indication of the intactness of chromosome 10, reflecting the absence of PTEN LOH. Indeed, when PTEN gene copy numbers were aligned to SCoR clusters, we observed an enrichment of tumors with normal (2N) PTEN copy number inside the SCoR cluster with increased chromosome 10 gene expression (Fig. 4a). To further confirm this hypothesis, we performed an unsupervised clustering on expression from all 791 chromosome 10 probesets from 188 glioblastomas (Fig. 4c). This recapitulated the findings from SCoR analysis. It identified a cluster of patients having elevated chromosome 10 gene expression, which was associated with normal PTEN copy numbers and highly enriched in long survival samples identified by SCoR (Fig. 4c). Based on PTEN copy number alone (normal vs. loss/deletion of PTEN) we could readily stratify glioblastoma patients into good and poor prognosis groups (Coxph *p* = 0.000568, data not shown). These results demonstrate the ability of SCoR analysis to infer prognostic gene copy number alterations from gene expression data. It also demonstrates the robustness of our method to identify prognostic signature associated with only a small percentage of patients (around 12% in TCGA glioblastomas). The result was repeatable on recently updated TCGA datasets having 437 glioblastomas (supplementary Fig. S2). These results further demonstrated the effectiveness of SCoR in reducing noises. If one considers chromosome 10 genes as real glioblastoma prognostic genes, then the median percentage of chromosome 10 genes found in all individual Coxph runs is 12.5% (ranging from 3.7% to 26%), whereas the percentage in the final SCoR result topped 30% (Fig. 4d).

### Identifying Patient Gender as a Prognostic Factor in Stage T1 Non-small Cell Lung Adenocarcinomas

**Figure 5:**
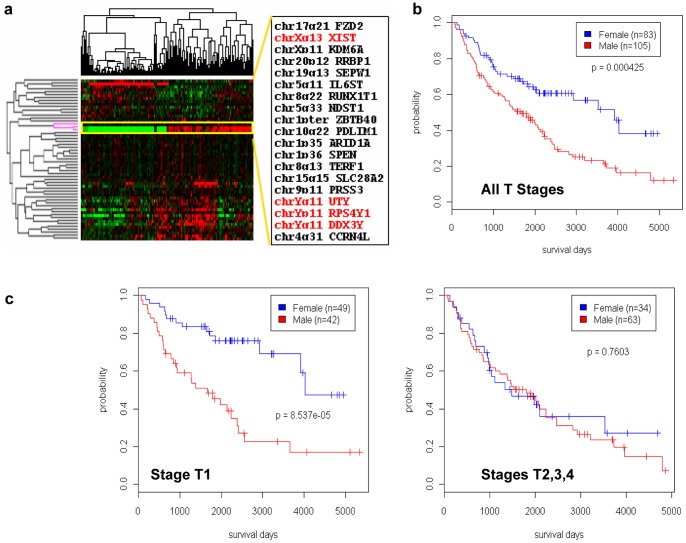
Identification of sex as a prognostic factor in one lung adenocarcinoma dataset. (a) cluster heatmap of gene expression using SCoR generated prognostic genes from DC2008-MI dataset annotated with gene symbols and chromosomal locations. (b) and (c) Kaplan Meier plot of patient survivals stratified by patient gender in all patients (b), and in stage T1 patients, stage T2, T3, T4 patients (c).

We applied SCoR to four lung adenocarcinoma datasets [14]. Interestingly, in one dataset (DCC2008-MI) the SCoR generated patient cluster was largely correlated with expression from three out of a total of 55 top candidate prognostic genes (Fig. 5a), two on chromosome Y (DDX3Y, RPS4Y1) and one on chromosome X (XIST). Since chromosome Y genes are only expressed in males, and the XIST transcript which is involved in X chromosome inactivation is only expressed in females, this result strongly argues that patient gender is related to patient survival. This was readily confirmed by Kaplan Meier analysis using clinical data on patient sex (Fig. 5, coxph *p* = 0.000425). When SCoR frequency cutoff was lowered to 50%, 5 chromosome Y genes could be picked up, out of overall 15 highly expressed chromosome Y genes found within this dataset. This demonstrates the sensitivity of our method to accurately identify only a small number of survival related genes. While we observed clear association of gender with survival in the DCC2008-MI dataset, we could not establish such a relationship using other three lung adenocarcinoma datasets from the same study [14]. To this end, we examined the difference in clinical data between DCC2008-MI and other three datasets and found that DCC2008-MI had a much higher percentage of patients with small sized tumors. We therefore further stratified DCC2008-MI patients based on both sex and AJCC tumor T stages. As shown in Fig. 5c, the effect of patient gender on survival could only be established in stage T1 tumors (size <3 cm, Coxph *p* = 8.537e−5), and there is no statistically significant difference in survival between male and female when tumor sizes exceeded 3 cm in stages T2, T3, T4 tumors (Coxph *p* = 0.7603) even within the DCC2008-MI dataset. These results are supported by previous reports [22]–[24] and are further supported by results from analyzing additional lung adenocarcinoma datasets (see supplemental Fig. S3), and again highlighted the importance to use compatible patient cohorts in survival analyses.

## Discussion

By incorporating random resampling (“jackknifing”) and unsupervised clustering techniques into traditional Coxph survival analysis, we developed the SCoR method which is more flexible and accurate in the survival analysis of large-scale microarray gene expression data. SCoR does not rely on a single, fixed training set, where we know results may be highly unstable, biased, and may sometimes miss real signals [12]. Instead, SCoR uses multiple training sets from jackknifing. Over the extensive resampling runs, overfit false positives generated from each run on individual training sets are diluted, whereas real signals are accumulated and enriched, making the output more accurate (e.g., Fig. 1c, 4d). Setting up of a frequency filter makes it more flexible to withstand system errors and noises. For example, a real prognostic gene may not be 100% times tested as prognostic when examined in a particular subset of patients. Also, microarray probes have different efficiency in gene detection, and microarray data quality may be sometimes poor and filled with more noise. In our experience, lowering the frequency filter to as low as 50% (i.e. half times to be prognostic in all runs) could still identify real signals (Table 1, Figs. 2, 4). Although lowering the filter will definitely introduce noise, this could be diluted out by the resampling procedure as discussed above.

A previous study by Liat Ein-Dor et al has pointed out that in breast cancer many genes are correlated with survival and the differences between these correlations are small [9]. Thus, even after hundreds of prognostic candidates are identified, it remains a challenge to properly rank these candidates, to distinguish noise from signals, and real signals from correlated surrogates (i.e. biological targets vs. biomarkers). Ranking based on *p* values or correlation coefficients is not convincing. Instead, we used unsupervised clustering to reveal any intrinsic structures associated with prognostic candidate gene expression, assuming subset samples with differential survival properties might have differential activation of signaling pathways and transcription programs that affect a group of genes. The validity of this approach is supported by the results in which top overlapping CDC genes from all SCoR runs on breast cancer datasets, which is likely to comprise a real prognostic signature, are highly enriched within the center of each SCoR gene cluster (Fig. 2).

Microarray studies are often aimed to find new biomarkers to assist known clinical parameters (e.g. tumor size, stage, sex) in predicting patient outcome. In fact, measurement of some of these factors, such as patient gender, ER level, or even gene copy number changes, could be recorded by microarray and therefore may appear as positive controls in survival analysis. However, few studies have reported such direct confirmations. Here, based on gene expression analysis alone, we identified ER/PR and Bcl-2 genes as good prognostic genes in one breast cancer dataset, sex related gene expression in one lung adenocarcinoma dataset, and elevated chromosome 10 gene expression (correlated with normal PTEN copy number) in two glioblastoma datasets. These results not only re-confirm the roles of ER/Bcl-2, sex, and PTEN copy number in breast cancer, lung cancer, and glioblastoma patient survival, respectively, but also serve as good validations of our analysis approach.

While the composition of various prognostic gene expression signatures for the same disease may differ, they may have similar prognostic power and utility in clinic [9], [25]. However, the functions of genes in the prognostic signature may relate to the biology of the disease at different levels, with the real prognostic gene expression signature faithfully reflects the biology of the disease. For example, an RB1 mutation related gene expression signature is the real signature behind retinoblastoma, and a p53 mutant related gene expression signature with changes in direct p53 transcriptional targets may be behind multiple cancer types caused by p53 mutation. Identifying such gene expression signature rather than the surrogates could help us understand the disease, find new therapeutic targets, and eventually improve patient survival. Our repeated finding of cell proliferation genes instead of the estrogen receptor gene as prognostic markers argues that targeting cell cycle division genes may be more beneficial in improving breast cancer patient survival.

The SCoR method can not only be used to analyze microarray data but also applied to any other high dimensional datasets, and to study association problems other than patient survival as well. For example, we have successfully used SCoR to analysis RNAseq data and identified existence of prognostic tumor subtypes using TCGA data (data not shown).

During the past decade, a large number of gene expression datasets were generated for many diseases using microarray technology and more data are being produced now on newer platforms such as RNAseq. Here we introduce a novel survival analysis method that is mathematically rigorous and more likely to offer biological insights into the disease. A revisit of existing gene expression databases and exploration of new generation of gene expression data using SCoR will greatly enhance the chance to make new discoveries.

## Supporting Information

Figure S1 **Identification of B-cell and T-cell markers as favorable prognostic signatures in highly proliferative breast cancers.** Cluster heatmaps of gene expression from SCoR generated prognostic genes were shown for subpopulations from GSE2034, GSE11121 (CDC-high), and NKI-295 (ER-negative). Blowup images show signatures containing B-cell marker genes (GSE2034, GSE11121) and T-cell marker genes (GSE2034, NKI295).
(PDF)

Figure S2 **Identification of chromosome 10 genes as good prognostic candidates in 437 glioblastoms from TCGA.** (a), cluster heatmaps of gene expression from SCoR generated prognostic genes with blowup box displaying chromosomal location, gene symbols. Patient survivals were plotted in blue or red (top and bottom quartile survivals in length). PTEN copy numbers were plotted in white, green, and red, representing normal copy number, copy loss, and deletion, respectively. (b), Kaplan Meier plot of patient survivals stratified by PTEN copy number status.
(PDF)

Table S1 **Gene expression datasets used in this study.**
(DOCX)

Table S2 **Cross-validation of prognostic gene expression signatures in different breast cancer datasets.** Different gene expression signatures were used to stratify patients and Coxph analysis *p* values were listed.
(DOCX)

Table S3 **Cross-validation of B- and T-cell gene expression signatures in different breast cancer datasets.** B- and T-cell signatures were used to stratify patients and Coxph analysis *p* values were listed.
(PDF)
