# Supplementary material for: Identification of Common Prognostic Gene Expression Signatures with Biological Meanings from Microarray Gene Expression Datasets
Source: PLoS One. 2012 Sep 21;7(9):e45894. doi: 10.1371/journal.pone.0045894 (PMC3448701; doi:10.1371/journal.pone.0045894)

Yao et al, Fig.S1, identification of B- and T-cell markers as favorable prognostic signatures in highly proliferative breast cancer.

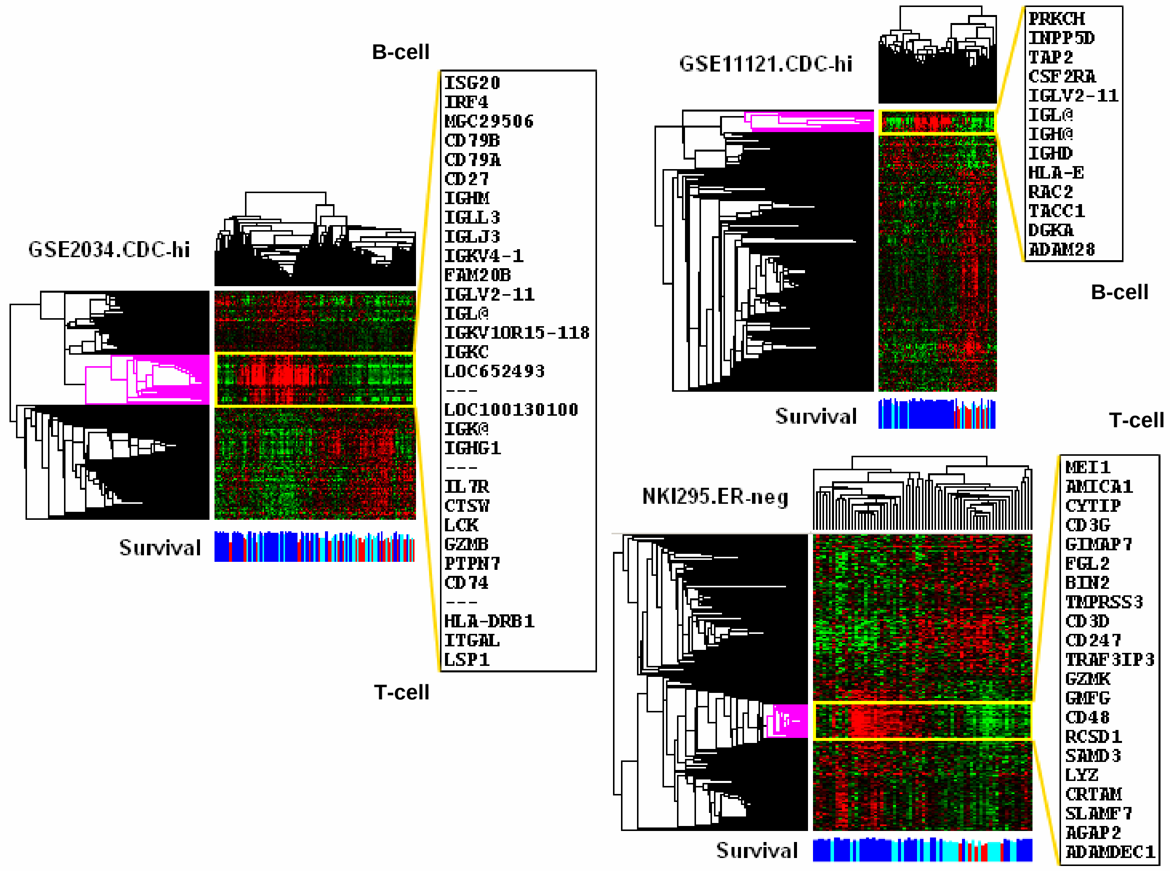

Supplement: Figure S1 — Identification of B-cell and T-cell markers as favorable prognostic signatures in highly proliferative breast cancers. Cluster heatmaps of gene expression from SCoR generated prognostic genes were shown for subpopulations from GSE2034, GSE11121 (CDC-high), and NKI-295 (ER-negative). Blowup images show signatures containing B-cell marker genes (GSE2034, GSE11121) and T-cell marker genes (GSE2034, NKI295). (PDF) [file pone.0045894.s001.pdf]
