# Supplementary material for: Identification of Common Prognostic Gene Expression Signatures with Biological Meanings from Microarray Gene Expression Datasets
Source: PLoS One. 2012 Sep 21;7(9):e45894. doi: 10.1371/journal.pone.0045894 (PMC3448701; doi:10.1371/journal.pone.0045894)

Yao et al, Fig.S2, Identification of chromosome 10 genes as good prognostic candidate genes in 437 glioblastomas from recently updated TCGA dataset

a

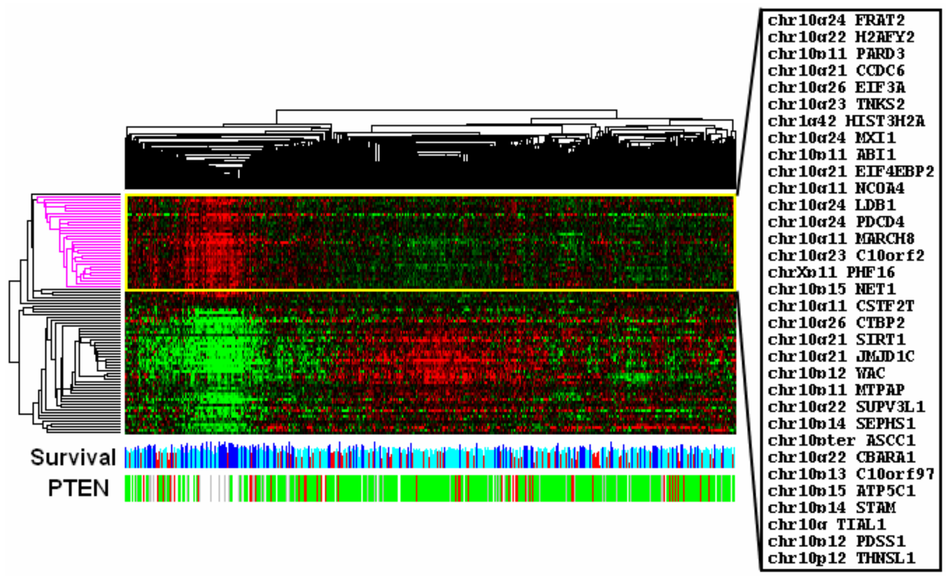

b

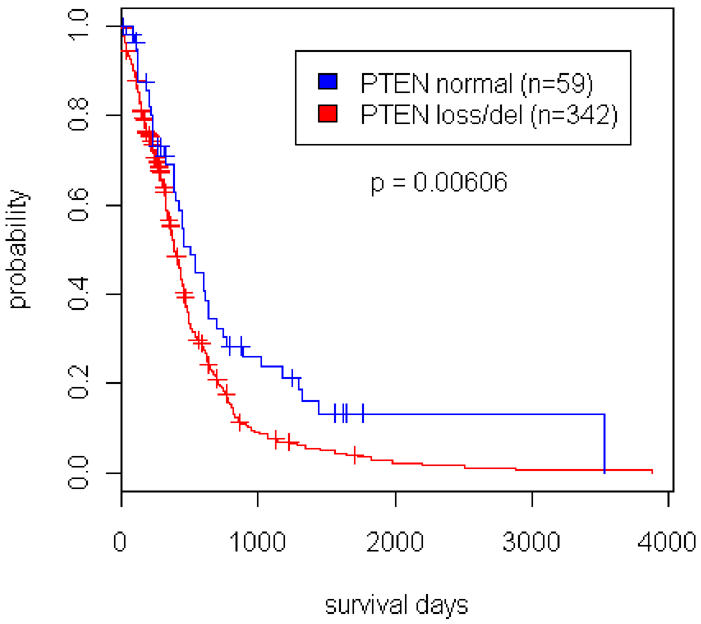

Supplement: Figure S2 — Identification of chromosome 10 genes as good prognostic candidates in 437 glioblastoms from TCGA. (a), cluster heatmaps of gene expression from SCoR generated prognostic genes with blowup box displaying chromosomal location, gene symbols. Patient survivals were plotted in blue or red (top and bottom quartile survivals in length). PTEN copy numbers were plotted in white, green, and red, representing normal copy number, copy loss, and deletion, respectively. (b), Kaplan Meier plot of patient survivals stratified by PTEN copy number status. (PDF) [file pone.0045894.s002.pdf]
