# Supplementary material for: Identification of Common Prognostic Gene Expression Signatures with Biological Meanings from Microarray Gene Expression Datasets
Source: PLoS One. 2012 Sep 21;7(9):e45894. doi: 10.1371/journal.pone.0045894 (PMC3448701; doi:10.1371/journal.pone.0045894)
Supplement: Table S1 — Gene expression datasets used in this study. (DOCX) [file pone.0045894.s003.docx]

Yao et al, Table S1, public datasets used in this study

| ***Dataset*** | ***Tumor type*** | ***Patient No.*** | ***Array platform*** | ***Reference*** |
| --- | --- | --- | --- | --- |
| NKI-295 | breast cancer | 294 | Rosetta 25K | [2] |
| GSE1456 | breast cancer | 159 | HG-U133A | [4] |
| GSE2034 | breast cancer | 286 | HG-U133A | [5] |
| GSE2990-KIU | breast cancer | 64 | HG-U133A | [6] |
| GSE3494 | breast cancer | 236 | HG-U133A | [3] |
| GSE7390 | breast cancer | 198 | HG-U133A | Desmedt et al, 2007 |
| GSE11121 | breast cancer | 200 | HG-U133A | [7] |
| GSE12093 | breast cancer | 136 | HG-U133A | Zhang et al, 2009 |
| GBM-TCGA-09 | glioblastoma | 188 | HT_HG-U133A | [21] |
| GBM-REMBRANDT | glioblastoma | 98 | HG-U133A_Plus2 |  |
| DC2008-MI | lung adenocarcinoma | 177 | HG-U133A | [14] |
| GSE4716 | non-small cell lung cancer | 50 | GF200/201 | Tomida et al, 2004 |
| GSE13213 | lung adenocarcinoma | 117 | Agilent-014850 | Tomida et al, 2009 |
| GSE25326 | non-small cell lung cancer | 69 | PMCC human 10.5K | Newnham et al, 2011 |
| GSE28582 | non-small cell lung cancer | 100 | HG-U133A_Plus2 | Micke et al, 2011 |

References not in the manuscript are listed below.

1. Desmedt C, Piette F, Loi S, Wang Y, Lallemand F et al. (2007) Strong time dependence of the 76-gene prognostic signature for node-negative breast cancer patients in the TRANSBIG multicenter independent validation series. Clin Cancer Res 13(11): 3207-3214.
2. Zhang Y, Sieuwerts AM, McGreevy M, Casey G, Cufer T et al. (2009) The 76-gene signature defines high-risk patients that benefit from adjuvant tamoxifen therapy. Breast Cancer Res Treat 116(2): 303-309.
3. Tomida S, Koshikawa K, Yatabe Y, Harano T, Ogura N et al. (2004) Gene expression-based, individualized outcome prediction for surgically treated lung cancer patients. Oncogene 23(31): 5360-70.
4. Tomida S, Takeuchi T, Shimada Y, Arima C, Matsuo K et al. (2009) Relapse-related molecular signature in lung adenocarcinomas identifies patients with dismal prognosis. J Clin Oncol 27(17): 2793-9.
5. Newnham GM, Conron M, McLachlan S, Dobrovic A, DoH et al (2011) Integrated mutation, copy number and expression profiling in resectable non-small cell lung cancer. BMC Cancer 11: 93.
6. Micke P, Edlund K, Holmberg L, Kultima HG, Mansouri L et al (2011) Gene copy number aberrations are associated with survival in histologic subgroups of non-small cell lung cancer. J Thorac Oncol 6(11): 1833-40.
