# Supplementary material for: Identification of Common Prognostic Gene Expression Signatures with Biological Meanings from Microarray Gene Expression Datasets
Source: PLoS One. 2012 Sep 21;7(9):e45894. doi: 10.1371/journal.pone.0045894 (PMC3448701; doi:10.1371/journal.pone.0045894)
Supplement: Table S2 — Cross-validation of prognostic gene expression signatures in different breast cancer datasets. Different gene expression signatures were used to stratify patients and Coxph analysis p values were listed. (DOCX) [file pone.0045894.s004.docx]

| ***signature*** | *NKI-295* | *GSE1456* | *GSE2034* | *GSE2990* | *GSE3494* | *GSE7390* | *GSE11121* | *GSE12093* |
| --- | --- | --- | --- | --- | --- | --- | --- | --- |
| NKI 70-gene | 5.17E-14 | 1.93E-03 | 1.23E-03 | **0.78** | 1.67E-03 | **0.81** | 3.97E-02 | 2.94E-02 |
| NKI-SCoR 69-gene | 4.67E-12 | 1.12E-02 | 1.10E-04 | **0.36** | 6.23E-03 | 1.32E-02 | 1.19E-02 | **0.08** |
| top overlap 85-gene | 1.12E-10 | 3.02E-06 | 4.32E-03 | 2.17E-01 | 1.85E-03 | 1.87E-04 | 9.11E-04 | 2.19E-02 |
| GSE1456 CDC 26-gene | 3.73E-10 | 2.78E-05 | 1.27E-03 | 2.44E-02 | 5.13E-04 | 4.52E-03 | 1.12E-02 | 9.73E-03 |
| GSE2034 CDC 27-gene | 2.95E-08 | 5.51E-05 | 9.49E-04 | 1.05E-02 | 1.01E-03 | 1.39E-03 | 4.66E-03 | 2.01E-02 |
| GSE3494 CDC 26-gene | 4.33E-12 | 1.49E-04 | 3.89E-03 | 3.69E-03 | 1.16E-03 | 1.70E-03 | 3.30E-03 | 4.59E-02 |
| GSE11121 CDC 26-gene | 1.68E-09 | 4.30E-05 | 7.76E-04 | **0.14** | 3.56E-04 | 2.61E-02 | 1.43E-02 | 1.35E-02 |
| GSE12093 CDC 14-gene | 5.11E-06 | 5.03E-04 | 1.69E-03 | **0.06** | 6.12E-05 | 7.16E-03 | 1.28E-03 | 1.15E-02 |

Yao et al, Table S2, cross validation of prognostic gene expression signatures in different breast cancer datasets
