# Supplementary material for: Identification of Common Prognostic Gene Expression Signatures with Biological Meanings from Microarray Gene Expression Datasets
Source: PLoS One. 2012 Sep 21;7(9):e45894. doi: 10.1371/journal.pone.0045894 (PMC3448701; doi:10.1371/journal.pone.0045894)
Supplement: Table S3 — Cross-validation of B- and T-cell gene expression signatures in different breast cancer datasets. B- and T-cell signatures were used to stratify patients and Coxph analysis p values were listed. (PDF) [file pone.0045894.s005.pdf]

Yao et al, Table S3, cross validation of B- and T-cell gene expression signatures in different breast cancer datasets

Gene Signature	Patient Group	GSE 1456	GSE 2034	GSE 2990	GSE 3494	GSE 7390	GSE 11121	GSE 12093	NKI 295
B-cell signature	CDC-high	0.329	**0.003**	0.381	0.276	**0.030**	**0.039**	0.091	0.623
	CDC-low	0.894	0.391	0.090	0.766	0.222	0.209	0.328	0.492
T-cell signature	CDC-high	0.051	**0.009**	0.436	**0.017**	**0.032**	**0.001**	0.060	0.163
	CDC-low	0.605	0.380	0.187	**0.046**	0.299	0.740	0.990	0.959
